# From the Oligonucleotide purUUpurU to Cytokine Storm, Elevated Blood Viscosity, and Complications of Coronavirus Disease 2019

**DOI:** 10.7759/cureus.25852

**Published:** 2022-06-11

**Authors:** Gregory D Sloop, Gheorghe A Pop, Joseph J Weidman, Liviu Moraru, John A St. Cyr

**Affiliations:** 1 Pathology, Idaho College of Osteopathic Medicine, Meridian, USA; 2 Cardiology, Radboud University Medical Center, Nijmegen, NLD; 3 Internal Medicine, Independent Researcher, Columbia, USA; 4 Anatomy, University of Medicine, Pharmacy, Science and Technology, Targu-Mores, ROU; 5 Cardiovascular Surgery, Jacqmar, Inc., Minneapolis, USA

**Keywords:** virus, rna, innate immunity, blood viscosity, cytokine storm syndrome, covid-19

## Abstract

Background

Coronavirus disease 2019 (COVID-19) can be associated with pathologic inflammation. The authors hypothesize that a high copy number of a purine-uridine-rich nucleotide motif is present in the genome of severe acute respiratory syndrome coronavirus 2 (SARS-CoV-2) and hyperactivates innate immunity.

Methods

The number of purine-uridine-uridine-purine-uridine (purUUpurU) motifs was counted in the genomes of SARS-CoV-2 and other single-strand RNA viruses. The nucleotides of SARS-CoV-2 in random order were used as a control.

Results

PurUUpurU occurred 2.8 times more often in the actual SARS-CoV-2 genome than the randomized genome. The number of purUUpurU motifs correlates with the potential severity of acute illness caused by these viruses, except for influenza A.

Conclusion

The large number of purUUpurU in SARS-CoV-2 may hyperactivate innate immunity, potentially causing the markedly increased concentrations of cytokines, acute phase reactants, and blood viscosity that can be seen in COVID-19.

## Introduction

Severe acute respiratory syndrome coronavirus 2 (SARS-CoV-2), the cause of coronavirus disease 2019 (COVID-19), has a single-strand RNA (ssRNA) genome around 29,900 bases long, making it among the largest ssRNA viruses that infect humans. Systemic inflammation (as opposed to local infection) caused by innate immunity begins with endocytosis of virus or viral components by inflammatory cells that are specialized to produce substantial amounts of cytokines [1]. Subsequently, breakdown of viral components in endolysosomes produces molecules called pathogen-associated molecular patterns (PAMPs), which activate innate immunity. The hydrolytic enzyme RNase T2 generates PAMPS after cleaving ssRNA between purine-uridine (purU) residues [2]. A second endonuclease, RNase 2, generates PAMPS by cleaving ssRNA between uridine-purine residues [3]. These PAMPS are recognized by toll-like receptor 8 (TLR8).

Binding of PAMPS to TLR8 upregulates the synthesis of tumor necrosis factor alpha (TNF-ɑ) and interleukin 6 (IL-6) [2]. TNF-ɑ is the “master regulator” of inflammatory cytokine production because of its early and broad role in mediating downstream cytokine production, including IL-6 [4]. Elevations of TNF-ɑ and IL-6 are strong independent predictors of poor prognosis in COVID-19 [5].

PurUUpurU motifs may contribute to inflammation in COVID-19

The genome of severe acute respiratory syndrome coronavirus (SARS-CoV-1) contains a large number of guanine-uridine rich sequences that stimulate expression in vitro of IL-6 and TNF-ɑ from the human acute monocytic leukemia cell line THP1, which expresses TLR8 but not toll-like receptor 7 [6]. The SARS-CoV-2 genome has 79% homology with that of SARS-CoV-1 [7]. Thus, the authors hypothesize that SARS-CoV-2 contains a large number of purU-rich sequences and can potentially hyperactivate TLR8-mediated inflammation, resulting in the marked elevations of TNF-ɑ, IL-6, fibrinogen, and blood viscosity observed in severe COVID-19.

The ssRNA oligomer UUGU is the minimal motif necessary for immunoactivity of the TLR8 agonist ssRNA40. Activation of TLR8 by this oligomer in monocytes, myeloid dendritic cells, and natural killer cells upregulates expression of several cytokines, including IL-6 and TNF-ɑ [8]. Hydrolysis of this oligomer by RNase T2 results in UUpur. Therefore, we determined the copy number of the motif 5’-purUUpurU-3’, which yields UUpur after hydrolysis with RNase T2, in the genomes of SARS-CoV-2, SARS-CoV-1, Middle East respiratory syndrome-coronavirus (MERS-CoV), human immunodeficiency virus-1 (HIV-1), hepatitis C virus (HCV), respiratory syncytial virus (RSV), and influenza A virus (IAV). As a control, we determined the number of purUUpurU in a hypothetical genome containing the nucleotides in the SARS-CoV-2 genome in a random order.

## Materials and methods

Extraction of nucleotide sequences from NIH NCBI

Genome files were downloaded from the National Center for Biotechnology Information (NCBI) of the National Institutes of Health website (https://www.ncbi.nlm.nih.gov/nuccore), either as a genbank or fasta format file containing every sequence for each virus. Several curations were performed to remove any misidentified sequences. The SARS-CoV-2 genome sequences were downloaded from the NCBI SARS-CoV-2 resources website (https://www.ncbi.nlm.nih.gov/sars-cov-2/).

PurUUpurU counting

Scripts were written using Python programming language (https://www.python.org/). The motif counting script uses SeqIO (Biopython; https://biopython.org/) and Regex functions to parse genomes and count the number of purUUpurU in each genome. The purines in purUUpurU can either be guanine or adenine, and some sequences may overlap, for instance, AUUAUUGU would count as two motifs, AUUAU and AUUGU. The strand present in viral particles (i.e., positive or negative) was analyzed. Results were stored in a comma-separated value file that was then imported into Microsoft Excel for statistical analysis and graphing.

Analysis of IAV

The IAV genome is composed of eight segments. A different number of each segment was available for analysis. The number of purUUpurU and total genome length were calculated by adding the mean number of purUUpurU and mean length of each segment. The coefficient of variation (CV) of the mean number of purUUpurU in the calculated total genome was determined by adding the standard deviations of each segment and dividing by the mean of the calculated genome length.

Randomized SARS-CoV-2 genome control

The library of randomized SARS-Cov-2 genomes was created using two scripts. First, nucleotides were extracted from a real SARS-CoV-2 genome, which was 29,903 nucleotides long. Second, a library of genomes containing these nucleotides in random order was created. This library was then analyzed with the counting script and processed similarly to real viruses.

## Results

**Figure 1:**
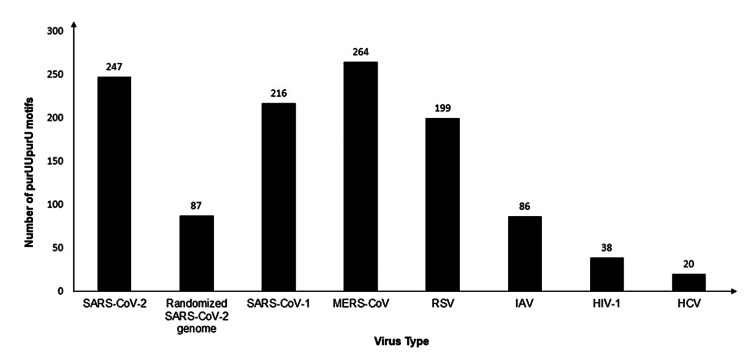
Graphic representation of the number of purUUpurU motifs in each genome. HCV, hepatitis C virus; HIV-1, human immunodeficiency virus-1; IAV, influenza A virus; MERS-CoV, Middle East respiratory syndrome-coronavirus; RSV, respiratory syncytial virus; SARS-CoV-2, severe acute respiratory syndrome coronavirus 2

All three coronavirus genomes contain a large number of purUUpurU (Table 1, Figure 1). PurUUpurU occurred 2.8 times more frequently in the actual SARS-CoV-2 genome than in the random genomes. RSV is the only non-coronavirus with more purUUpurU than the random SARS-CoV-2 genome. Of the viruses with many purUUpurU, RSV has the most variability in the number of this motif, with a CV of 6.0%. HIV-1 has the most variability in genome length (CV = 3.0%). Sequences that contained overlapping motifs, such as AUUAUUGU, were rare and had a negligible impact on the number of purUUpurU.

**Table 1:** Analysis of viral genomes for purUUpurU *This is the number of segments analyzed, not complete genomes. CV, coefficient of variation; HCV, hepatitis C virus; HIV-1, human immunodeficiency virus-1; IAV, influenza A virus; MERS-CoV, Middle East respiratory syndrome-coronavirus; RSV, respiratory syncytial virus; SARS-CoV-2, severe acute respiratory syndrome coronavirus 2

Virus	Number of purUUpurU		Genome size (number of nucleotides)		Number of genomes analyzed
	Mean	CV	Mean	CV	
SARS-CoV-2	247	0.80%	29.810	0.60%	30,644
Randomized SARS-CoV-2 genome	87	11.00%	29.903	0	30,644
SARS-CoV-1	216	0.50%	29.688	0.40%	127
MERS-CoV	264	1.50%	30.110	0.10%	470
RSV	199	6.00%	15.165	0.50%	1615
IAV	86	9.30%	13.588	NA	29,703*
HIV-1	38	13.00%	9210	3.00%	3173
HCV	20	25.00%	9415	1.00%	16,688

Because all 30,644 randomized SARS-CoV-2 genomes were 29,903 nucleotides long, the CV of the mean number of nucleotides in the random genome is 0. Because a different number of each segment in the IAV genome was analyzed, the CV of the mean of its genome length was not calculated. The standard deviation of the mean number of purUUpurU in each of the eight segments in the IAV genome was 1.

## Discussion

PurUUpurU is a pathogenic factor in ssRNA viruses

The substantial number of purUUpurU in SARS-Cov-2 and other coronaviruses could contribute to the potential severity of the acute illness caused by these viruses, which can be fatal. Except for IAV, there is a correlation between the potential severity of the acute illness caused by these viruses and the number of purUUpurU in their genomes. RSV has substantial number of purUUpurU and causes a potentially fatal illness in children younger than one year and adults ≥ 50 years. HIV-1 has fewer purUUpurU and causes a nonfatal flu-like syndrome in 50-70% of acute infections. Acute infection with HCV is usually asymptomatic and mild when symptomatic, consistent with the even smaller number of purUUpurU in its genome.

Given the low copy number of purUUpurU in its genome, IAV must cause inflammation by a pathway not involving TLR8. Viral structural proteins are also PAMPs and can activate inflammation via other toll-like receptors. In addition, retinoic acid-inducible gene-1 (RIG-1) and nucleotide-binding oligomerization domain-like receptor pyrin domain-containing 3 (NLRP3) are cytoplasmic receptors that are expressed widely and can sense IAV [1].

The large CV in the mean number of purUUpurU in the RSV genomes may be due to the existence of two strains of RSV, A and B. These differ in the severity of disease they cause, which may correlate with the number of purUUpurU in their respective genomes. We are exploring this possibility. The large CV of the mean number of purUUpurU in HIV-1, IAV, and HCV is due to the existence of multiple subtypes of these viruses.

The ability of innate immunity to limit replication of SARS-CoV-2 is limited because the virus elicits weak expression of interferon types I and III, cytokines that reduce viral replication [9]. In the absence of a robust response with these interferons, sustained viral replication can generate substantial numbers of purUUpurU. Excessive stimulation of innate immunity by the large quantity of purUUpurU in SARS-CoV-2 may be more important in causing cytokine storm syndrome in COVID-19 than putative dysregulation of cytokine expression by the host immune system.

Effects of hyperactivation of TLR8

**Figure 2:**
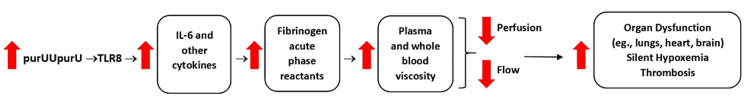
The pathogenesis of complications of COVID-19 caused by purUUpurU. pur, purine; U, uridine; TLR, toll-like receptor; IL, interleukin

Because cytokine storm syndrome has been reported in all three of these coronaviral diseases [10], hyperstimulation of innate immunity by purUUpurU may be a common pathway in the development of this complication. Identification of this stimulus for cytokine production in COVID-19 allows informed conjecture about how marked elevations of cytokines cause pathology (Figure 2). Hyperstimulation of TLR8 by purUUpurU will greatly increase plasma concentrations of IL-6. This cytokine upregulates expression of acute phase reactants, such as fibrinogen and ferritin, and downregulates others, such as albumin. Increased fibrinogen concentrations elevate plasma viscosity and augment erythrocyte aggregation. Decreased albumin concentrations decrease erythrocyte deformability and increase erythrocyte aggregation. These changes make inflammation a state of elevated blood viscosity. The fibrinogen concentration and plasma viscosity can be higher in COVID-19 than in any other commonly diagnosed disease, 14 g/L and 4.2 cP, respectively [11]. In a study of 15 COVID-19 patients in an intensive care unit, blood viscosity was 23% greater than in 23 patients studied more than eight weeks after infection [12].

Complications of elevated blood viscosity

Viscosity describes the resistance of a fluid to flow. Honey is more viscous than water. A change in blood viscosity causes a threefold inverse change in blood flow [13]. Decreased blood flow increases the risk of thrombosis and decreases tissue perfusion. In the 19th century, Virchow identified sluggish blood flow as a risk factor for thrombosis [11]. Sluggish blood flow is simply the expression of increased blood viscosity. It downregulates shear-mediated expression of molecules with antiplatelet activity such as prostacyclin and nitric oxide, decreases influx of antithrombotic proteins, and allows the accumulation of activated coagulation factors. COVID-19, SARS, and MERS are associated with an increased risk of thrombosis [14], and COVID-19 is a risk factor for myocardial infarction and ischemic stroke [15]. An increased risk of myocardial infarction is noted with viral, bacterial, and parasitic infections as well as immunization [13]. Elevated blood viscosity is an important contributor to the phenomenon known as “thromboinflammation.”

Increased blood viscosity decreases the transportation of oxygen and metabolic substrates to all tissue [16], reducing their ability to synthesize adenosine triphosphate and other molecules and perform mechanical work. Reduced pulmonary blood flow causes global ventilation-perfusion (V/Q) mismatch resulting in the condition commonly called “silent hypoxemia” or “happy hypoxia.” Increased blood viscosity does not cause dyspnea because it is increased, not decreased blood flow that activates the perivascular stretch receptors in the lung that initiate the sensation of dyspnea [11].

Increased pulmonary vascular resistance in conjunction with decreased myocardial perfusion results in right ventricular dilatation, which is the most common echocardiographic abnormality in hospitalized COVID-19 patients [17]. Decreased myocardial perfusion also causes left ventricular diastolic dysfunction, the second most common echocardiographic abnormality in that study. Mismatch of oxygen supply and demand due to inadequate perfusion caused by elevated blood viscosity also results in biochemical and radiographic evidence of myocarditis in COVID-19 [11]. Increased blood viscosity also causes myocarditis following immunization for COVID-19 [18].

Decreased perfusion of the brain causes abnormalities on susceptibility-weighted imaging (SWI). This is the most common lesion seen on magnetic resonance imaging in hospitalized COVID-19 patients [19]. This imaging sequence is also called “blood oxygen level dependent” (BOLD) venography because it is sensitive to deoxyhemoglobin. Most lesions involved white matter because decreased perfusion impairs the ability of oligodendrocytes to synthesize myelin.

Those who suffer from obesity, hypertension, diabetes mellitus, chronic obstructive pulmonary disease and congestive heart failure are at high risk for a poor outcome from COVID-19. These patients often have elevated blood viscosity at baseline, typically, as a result of impaired erythrocyte deformability. The additional elevation of blood viscosity imposed by a hyperinflammatory state such as COVID-19 increases the risk of complications in these patients.

## Conclusions

The large number of purUUpurU in SARS-CoV-2 may hyperactivate innate immunity, potentially causing the markedly increased concentrations of cytokines, acute phase reactants, and blood viscosity that can be seen in COVID-19.
